# 2143. *in-vitro* activity of Bacteriophages against Methicillin-Resistant *Staphylococcus aureus* Biofilms

**DOI:** 10.1093/ofid/ofad500.1766

**Published:** 2023-11-27

**Authors:** Krupa Parmar, Waqas Chaudhry, Joseph Fackler, John M Sowers, Aravinda Vadlamudi, Kerryl E Greenwood-Quaintance, Robin Patel

**Affiliations:** Mayo Clinic, ROCHESTER, Minnesota; Adaptive Phage Therapeutics Inc., Gaithersburg, Maryland; Adaptive Phage Therapeutics Inc., Gaithersburg, Maryland; Adaptive Phage Therapeutics Inc., Gaithersburg, Maryland; Adaptive Phage Therapeutics, Frederick, Maryland; Mayo Clinic, ROCHESTER, Minnesota; Mayo Clinic, ROCHESTER, Minnesota

## Abstract

**Background:**

Methicillin-resistant *Staphylococcus aureus* (MRSA) biofilms can be resilient to the human immune system and antibiotics, a challenge augmented by acquired antimicrobial resistance. Phage therapy is being evaluated for biofilm-associated infections.

**Methods:**

Here, model phages SaMD07ø1 and SaRB105030ø5 were evaluated against their hosts SaMD07 and SaRB105030, respectively, grown as biofilms in 96-well plates, as biofilms on glass beads and planktonically. Trypticase soy broth (TSB) and phosphate buffered saline (PBS) were evaluated as testing media (Figure 1). Biofilms were incubated with phages in the presence of media containing tetrazolium dye in 96-well plates and assessed for hold times (delays in reaching bacterial log-phase metabolic activity compared to controls) over 48 hours (h) using a Biolog Omnilog® system and by assessing colony forming units (CFUs). Subsequently, using the beads biofilm assay in TSB medium performed on the Omnilog® (BBTO) (Figure 2), test phage SaMD22ø1 was evaluated in quintuplicate against biofilms formed by MRSA isolates IDRL-6130, IDRL-6927, IDRL-6987, IDRL-7381, IDRL-7542 and IDRL-10947, all recovered from periprosthetic joint infection. Test phage K was evaluated against GFP-labelled MRSA AH1263 using BBTO method and simultaneously by fluorescence microscopy.

**Figure 1.**
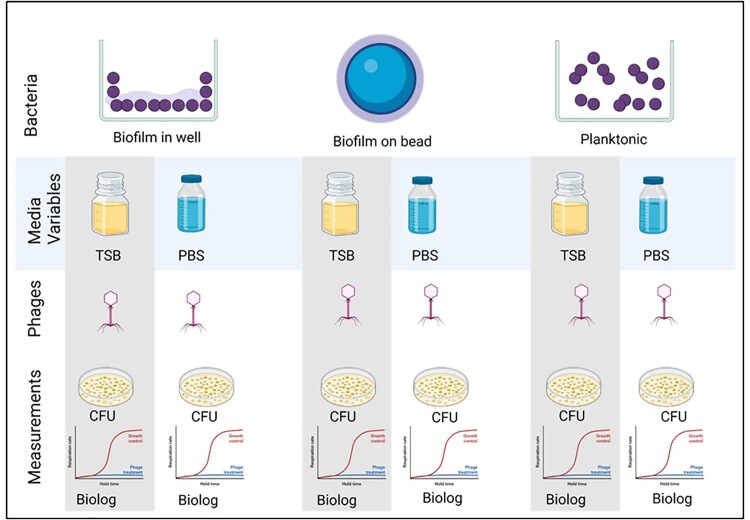
Biofilm phage susceptibility testing methods evaluated (created with BioRender.com)

**Figure 2.**
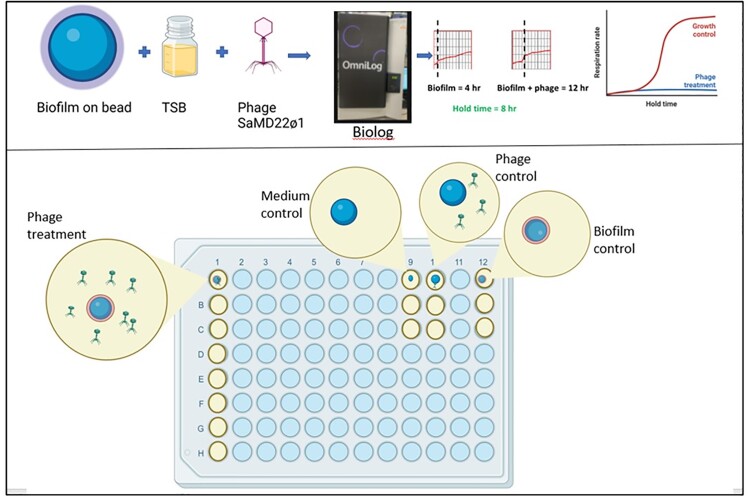
Biofilm phage susceptibility testing using biofilms grown on beads assessed in TSB medium using the Omnilog® (BBTO) method with phage SaMD22ø1.

**Results:**

A comparison of result of testing of biofilms in wells and on beads and against planktonic bacteria is shown in Figure 3. Phage SaMD07ø1 showed no growth at 4 h; whereas phage SaRB105030ø5 showed ∼1-log decrease in CFUs at 4 h and ∼3-log decrease in CFUs at 8 h (Figure 3). BBTO hold-times of 37, 28 and 15 h were observed for phages SaMD07ø1, SaRB105030ø5 and phage K respectively. Phage SaMD22ø1 had an average hold time of 9 h and showed hold times >6 hours for all isolates tested (Figure 4). Microscopic inspection showed eradication of GFP-labelled MRSA biofilm by phage K (Figure 5).

**Figure 3.**
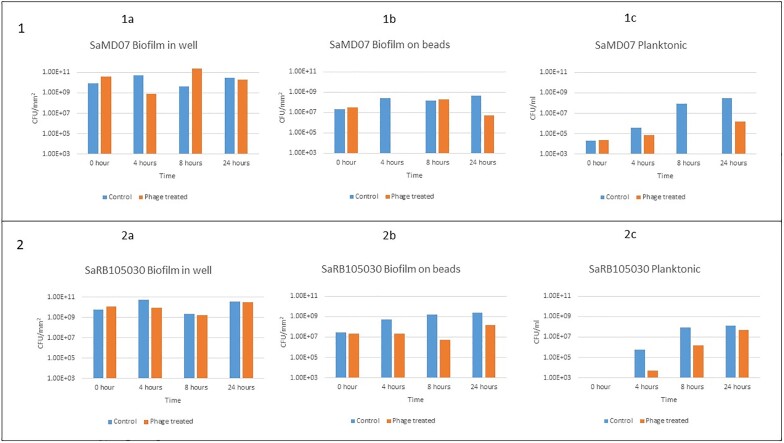
Results of quantitative culture of biofilms treated with phages SaMD07ø1 and SaRB105030ø5 in TSB medium. Panel 1 shows results of phage SaMD07ø1 with biofilms grown in wells (1a), on beads (1b) and planktonically (1c). Panel 2 shows results of phage SaRB105030ø5 with biofilms grown in wells (2a), on beads (2b) and planktonically (2c).

**Figure 4.**
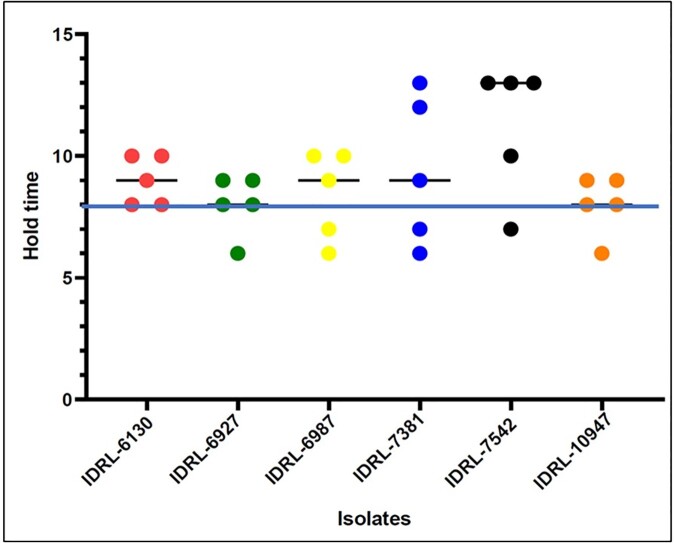
Activity of phage SaMD22ø1 against biofilms formed by six MRSA strains determined using the Omnilog® (BBTO) method.

The solid blue line indicates a hold time of 8 hours.

**Figure 5.**
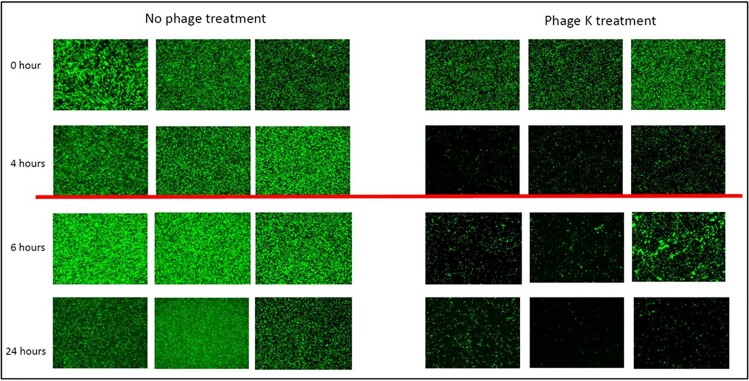
Efficacy of Phage K to efficiently eradicate GFP-labelled MRSA strain AH1263 observed under the fluorescence microscope at 40X magnification.

The authors are thankful to Dr. Alexander Horswill at University of Colorado for providing the GFP-labelled MRSA strain.

**Conclusion:**

In conclusion, BBTO is a promising method for testing biofilm phage susceptibility although clinical correlation and testing of larger numbers of phage and bacterial isolates are needed.

**Disclosures:**

**Joseph Fackler, n/a**, Adaptive Phage Therapeutics, Inc: Employee|Adaptive Phage Therapeutics, Inc: Stocks/Bonds **John M. Sowers, n/a**, Adaptive Phage Therapeutics: Employee|Adaptive Phage Therapeutics: Stocks/Bonds **Robin Patel, MD**, Abbott Laboratories: Advisor/Consultant|Adaptive Phage Therapeutics: Grant/Research Support|Adaptive Phage Therapeutics: Mayo Clinic has a royalty-bearing know-how agreement and equity in Adaptive Phage Therapeutics.|BIOFIRE: Grant/Research Support|CARB-X: Advisor/Consultant|ContraFect: Grant/Research Support|Day Zero Diagnostics: Advisor/Consultant|HealthTrackRx: Advisor/Consultant|Mammoth Biosciences: Advisor/Consultant|Netflix: Advisor/Consultant|Oxford Nanopore Technologies: Advisor/Consultant|PhAST: Advisor/Consultant|See details: Patent on Bordetella pertussis/parapertussis PCR issued, a patent on a device/method for sonication with royalties paid by Samsung to Mayo Clinic|See details: continued, patent on an anti-biofilm substance issued|TenNor Therapeutics Limited: Grant/Research Support|Torus Biosystems: Advisor/Consultant|Trellis Bioscience, Inc.: Advisor/Consultant

